# Impact of Early Empiric Antibiotic Regimens on the Gut Microbiota in Very Low Birth Weight Preterm Infants: An Observational Study

**DOI:** 10.3389/fped.2021.651713

**Published:** 2021-05-31

**Authors:** Hung-Yang Chang, Jen-Shiu Chiang Chiau, Yu-Hsuan Ho, Jui-Hsing Chang, Kun-Nan Tsai, Chia-Yen Liu, Chyong-Hsin Hsu, Chia-Ying Lin, Mary Hsin-Ju Ko, Hung-Chang Lee

**Affiliations:** ^1^Department of Pediatrics, MacKay Children's Hospital, Taipei, Taiwan; ^2^Department of Medicine, MacKay Medical College, New Taipei City, Taiwan; ^3^Department of Medical Research, MacKay Memorial Hospital, Taipei, Taiwan; ^4^Life Science, Delta Research Center, Delta Electronics Incorporation, Taipei, Taiwan

**Keywords:** preterm, very low birth weight infants, antibiotics, microbiota, microbiome

## Abstract

Frequent use of antibiotics in preterm infants disturbs their gut microbial balance. In this preliminary observational study, we investigated the effect of different antibiotic regimens, administered during the first week of life, on microbial composition and diversity in very low birth weight (VLBW) preterm infants. We performed fecal sampling of breastfed VLBW infants on days 7, 14, and 30. After excluding stool samples from infants who received probiotics or who were administered antibiotics beyond the age of 7 days, we compared gut microbiota profiles between infants receiving a combination of ampicillin and gentamicin for 3 days (AG group, *n* = 10) and those receiving a combination of ampicillin and cefotaxime for 7 days (AC group, *n* = 14) using 16S ribosomal DNA community profiling. We also assessed the changes over time in each group. Compared to the AG group, *Enterococcus* species were significantly more abundant in the AC group (*P* = 0.002), especially in 7-day samples (12.3 vs. 0.6%, respectively, *P* = 0.032). No difference was observed at phylum and genus level over time within each group. Species richness in the AC group decreased significantly in the 14-day (*P* = 0.038) and 30-day (*P* = 0.03) samples compared to that in the 7-day sample. The same was observed for microbial evenness; in contrast, no significant difference in Shannon index and beta-diversity was detected between the two groups. Controlling for relevant confounding variables did not change the results. In conclusion, different antibiotic regimens affect the early development of gut microbiota in VLBW preterm infants. Prolonged use of ampicillin and cefotaxime might result in overabundance of *Enterococcus*. However, given that no significant differences were observed in 1-month samples, bacterial genera appear to continue colonizing the gastrointestinal tract despite previous exposure to antibiotics. The clinical relevance of these findings should be elucidated by further studies.

## Introduction

The gut microbiome plays a vital role in promoting overall health, and the perinatal period is considered critical for the development of infant gut microbiota. A healthy gut microbiota contributes significantly to the development of a healthy immune system, digestive functions, and neurological functions (1–3). The gut microbiota is highly dynamic during early infancy and is affected by several host and environmental factors, including gestational age, mode of delivery, type of feeding, administration of probiotics, living environment, and medical interventions (1–6). The onset and progression of several infectious diseases, chronic inflammatory bowel disease, allergies, obesity, and diabetes, are associated with disruption of the gut microbiota (2, 7).

The gut microbiota of preterm neonates, as compared to full-term neonates, is unique because it is characterized by delayed colonization of common bacterial genera and an abundance of pathogens (8, 9). Owing to concerns about a relatively immature immune system that may not elicit an optimal response to infection, preterm infants are generally prescribed empiric antibiotic therapy in neonatal intensive care units (NICUs) (10). Prolonged use or upgrade of antibiotics is very common following peripartum issues, such as prolonged rupture of membranes and suspected chorioamnionitis in mothers or non-specific symptoms and signs of infection in neonates. Previous studies showed that antibiotic overuse in early life could impair initial microbial colonization (11–14), which might contribute to subsequent adverse outcomes in preterm infants, such as late-onset sepsis (LOS), necrotizing enterocolitis (NEC), and even death (15–19).

Despite an increased understanding of how antibiotic treatment influences microbiota development in preterm infants, the impact of different antibiotic exposure on preterm microbial community is not well-studied. This preliminary observational study's primary goal was to investigate the effect of different antibiotic regimes, administered during the first week of life, on the development of gut microbiota in very low birth weight (VLBW, birth weight <1,500 g) preterm infants.

## Materials and Methods

### Study Subjects

This prospective cohort observational study was conducted at the NICU of MacKay Children's Hospital from May 2017 until May 2018. The study was supported by a grant (MMH-106-69) from MacKay Memorial Hospital. Eligible subjects were VLBW preterm infants who received their mothers' milk. Exclusion criteria included infants with major congenital anomalies or malformations, those receiving nothing by mouth for ≥7 days or using probiotics during the study period, and those administered antibiotics after the age of 7 days. The study was approved by the Institutional Review Board of MacKay Memorial Hospital, and was performed in accordance with the 1964 Declaration of Helsinki and its later amendments (IRB number: 17MMHIS026e). Infants were recruited after written informed consent had been obtained from their parents.

Infants were given antibiotic treatment on clinical suspicion of a bacterial infection after analyzing blood cultures. Early empiric antibiotic use consisted of ampicillin plus gentamicin or ampicillin plus cefotaxime. Infants were divided into two groups: those who received 3 days of combination treatment with ampicillin plus gentamicin (AG group), and those who received 7 days of ampicillin plus cefotaxime (AC group). Treatment doses were based on existing guidelines and were adjusted according to patients' gestational age and birth weight. Antibiotic combinations and treatment duration were determined by the attending physicians based on the perceived risk of infection. Ampicillin and third-generation cephalosporins were administered to patients with high risk of infection over a period of 1 week as per our NICU protocols. Medical records were reviewed to identify pertinent infants and maternal factors, as well as feeding patterns.

### Sample Collection and DNA Extraction

Freshly evacuated feces from soiled diapers were collected at 7, 14, and 30 days by medical staff. All samples were frozen at −80°C until further processing. For the extraction of fecal DNA, the QIAmp® DNA stool mini kit (Qiagen, Germany) was used according to the manufacturer's instructions.

### PCR Amplification and Illumina MiSeq

The V3-V4 region of the 16S rRNA gene was amplified by PCR using universal primers (16S Amplicon PCR Forward Primer: 5′ TCGTCGGCAGCGTCAGATGTGTATAAGAGACAGCCTACGGGNGGCWGCAG; 16S Amplicon PCR Reverse Primer: 5′ GTCTCGTGGGCTCGGAGATGTGTATAAGAGACAGGACTACHVGGGTATCTAATCC) (20). The PCR was performed in a total volume of 25 μL containing 0.2 μM of each primer and 12.5 μL of 2X KAPA HiFi HotStart ReadyMix (Roche, South Africa). PCR conditions were as follows: 95°C for 3 min; 25 cycles at 95°C for 30 s, 55°C for 30 s, and 72°C for 30 s; and a final extension at 72°C for 5 min in a Veriti® 96-well thermal cycler (Applied Biosystems, Singapore). The purification step was carried out using AMPure XP beads (Beckman Coulter, USA). Dual indices and Illumina sequencing adapters were attached to the PCR products using the Nextera XT Index Kit for each library. The treated products were quantified on a NanoDrop ND2000 spectrophotometer (Thermo Scientific, USA), Qubit dsDNA HS Assay Kit (Invitrogen, USA), and Labchip GX Touch 24 (Applied Biosystems, USA). Then, the amplicons were pooled together in equimolar quantities and paired-end sequenced (2 × 300 bp) on an Illumina MiSeq platform according to standard protocols.

### Bioinformatics Analysis of Microbiota

To analyze bacterial communities, raw sequence reads were assessed for quality and then analyzed by QIIME (21). Each sample yielded more than 100,000 reads after pre-processing in USEARCH 6.1, which included a chimera check and merging of chimera-checked sequences. To pick and cluster the operational taxonomic units (OTUs) in each sample, we used the GreenGenes bacterial reference database with a threshold of 97% sequence identity, thus allowing for maximal taxonomic specificity. Based on taxonomic analysis and species annotation, we generated relative abundance profiles of OTUs for all samples. Differences in community structure were analyzed at the phylum and genus levels; microbial richness and evenness were assessed by the Chao1 and Simpson's Evenness (Simpson's E) indices, respectively. The Shannon index was computed as a measure of alpha-diversity for each individual. To assess differences across groups (beta-diversity), we compared the AC and AG groups by principal component analysis (PCA) and visualized the results in R version 3.5 (https://www.r-project.org/).

### Statistical Analyses

Patient characteristics were assessed using a two-sample *t*-test for continuous variables and Fisher's exact test for categorical variables. To determine significant differences across samples, the Kruskal-Wallis test was conducted for multiple comparisons followed by Dunn's *post-hoc* test using Benjamini-Hochberg for correction. Composition and alpha-diversity were compared between the two groups at three time points and over time within a group. Permutational multivariate analysis of variance (PERMANOVA) was performed to assess the effect of relevant confounding factors, including maternal antibiotics use, preterm rupture of membranes, birth weight, start of feeding, and chronic lung disease. Analysis of similarities (ANOSIM) was used for comparison of PCA results between groups. *P* < 0.05 was considered statistically significant.

## Results

### Demographic Characteristics

Eighty-four breastfed VLBW infants were admitted to our NICU during the study period. After excluding infants who were administered probiotics (*n* = 40) or antibiotics after 1 week of age (*n* = 20), serial stool samples of the final cohort (*n* = 24) were analyzed. Based on exposure to different early postnatal antibiotic therapy, the 24 cases were divided into two groups: AG group (*n* = 10) and AC group (*n* = 14). The reasons for ampicillin-cefotaxime prescription included maternal chorioamnionitis (*n* = 4), increased levels of maternal C-reactive protein (*n* = 5), and infant leukopenia or leukocytosis (*n* = 5). Gestational age and birth weight were 30.0 ± 2.5 weeks and 1,286 ± 190 g in the AG group, and 28.9 ± 2.5 weeks and 1,099 ± 280 g in the AC group. Although there were no statistically significant differences among demographic and clinical characteristics between these two groups, infants in the AC group were more premature and carried a higher risk of infections than those in the AG group (Table 1).

**Table 1:** Demographic and clinical characteristics of study subjects.

	**AG group^a^ (*N* = 10)**	**AC group^a^ (*N* = 14)**	***P*-value**
Maternal antibiotics use	8	10	0.75
Prenatal steroid use	9	14	0.71
PROM >18 h	4	9	0.34
Cesarean delivery	7	12	0.36
Gestational age (week)^b^	29.0 (28.0–31.3)	29.0 (27.5–31.0)	0.74
Birth weight (gram)^b^	1,324 (1,223–1,405)	1,138 (846–1,388)	0.09
Male	6	6	0.42
SGA	4	10	0.13
Multiple births	4	1	0.06
APGAR score 5 mins <7	0	1	0.40
Start feeding (day)^b^	2.0 (2.0–3.3)	4.0 (2.0–7.0)	0.07
Reach full feeding (day)^b^	16.0 (10.8–24.5)	21.0 (14.8–29.5)	0.21
RDS need surfactant	5	8	0.49
Days on Oxygen (day)^b^	12.0 (4.3–49.3)	42.0 (3.5–70.5)	0.38
CLD	2	8	0.08
Any IVH	0	1	0.40
ROP need treatment	0	4	0.40
PDA need treatment	3	1	0.94
Total hospitalization days^b^	47.5 (40.8–63.5)	62.0 (44.0–76.0)	0.11

*No cases of sepsis and necrotizing enterocolitis in both groups*.

a *The values represent the number of participants, unless otherwise specified*.

b *Values are expressed as median (IQR)*.

*AG, ampicillin-gentamicin group; AC, ampicillin-cefotaxime group; PROM, preterm rupture of membranes; SGA, small for gestational age; RDS, respiratory distress syndrome; CLD, chronic lung disease; IVH, intraventricular hemorrhage; ROP, retinopathy of prematurity; PDA, patent ductus arteriosus*.

### Fecal Samples

#### Analysis of Microbiota Composition

**Figure 1:**
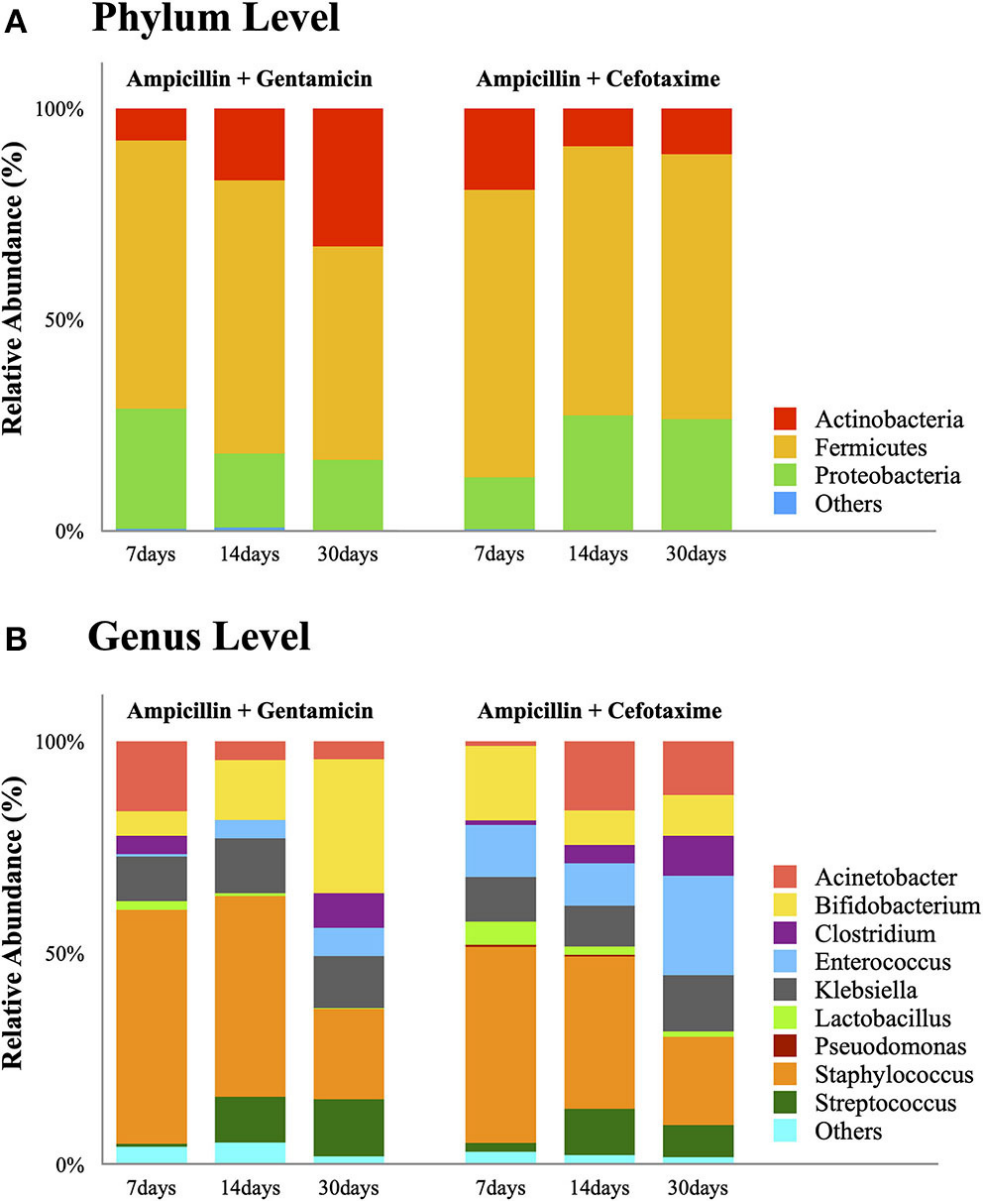
Bar plots showing relative abundance of the most dominant bacterial communities in the AG and AC groups on days 7, 14, and 30. **(A)** Relative abundance at the phylum level. **(B)** Relative abundance at the genus level. AG, ampicillin-gentamicin group; AC, ampicillin-cefotaxime group.

Five stool samples (two 14-day samples in both groups, one 30-day sample in the AG group) could not be obtained due to absence of bowel movement at that time point. Valid sequence data (passing quality control) generated 67 samples. Overall microbiota composition of each group at phylum and genus level is illustrated in Figure 1.

##### Phylum Level

Detailed phylum-level analysis revealed no difference in the distribution of predominant phyla among both groups at any time. Although microbial abundance decreased with increasing age, *Firmicutes* was the most abundant phylum in both groups and remained dominant until day 30 (50.6% in the AG group and 62.7% in the AC group). In the AG group, the proportion of *Actinobacteria* increased over time (7.6, 17.1, and 32.7% at 7, 14, and 30 days, respectively); whereas that of *Proteobacteria* decreased (28.4, 17.5, and 16.7% at 7, 14, and 30 days, respectively). In contrast, in the AC group, the proportions of these two major bacterial phyla showed the opposite trend (*Actinobacteria*: 19.3, 9.0, and 10.9% at 7, 14, and 30 days, respectively; and *Proteobacteria*: 12.3, 27.2, and 26.3% at 7, 14, and 30 days, respectively). No statistically significant difference in the observed bacterial phyla was detected over time within any of the groups (all *P* > 0.05).

##### Genus Level

Except for *Enterococcus*, no statistical difference in bacterial composition at the genus level was observed between the AG and AC groups at any time or over time within any of the groups. Compared to the AG group, *Enterococcus* was significantly more abundant in the AC group (*P* = 0.002), especially in the 7-day sample (12.3 vs. 0.6%, respectively, *P* = 0.032). Such low abundance of *Enterococcus* in the AG group persisted even after controlling for relevant confounding factors. Although the relative abundance of each bacterium at genus level displayed no statistically significant difference over time within any group (all *P* > 0.05), the proportion of *Enterococcus* increased in both groups (AG group: 0.6, 4.4, and 6.7% at 7, 14, and 30 days, respectively; AC group: 12.3, 13.1, and 23.5% at 7, 14, and 30 days, respectively). In contrast, the genus *Bifidobacterium* increased over time in the AG group (5.8, 14.1, and 31.6% at 7, 14, and 30 days, respectively), but decreased in the AC group (17.6, 8.1, and 9.7% at 7, 14, and 30 days, respectively). Indeed, *Bifidobacterium* became a dominant member of the community in the AG group at 30 days after birth (31.6%). Although the proportion decreased over time, *Staphylococcus* remained the top genus in both groups on days 7 and 14 (AG group: 55.3, 47.5, and 21.4% at 7, 14, and 30 days, respectively; AC group: 46.4, 36.0, and 20.9% at 7, 14, and 30 days, respectively). The abundance of *Lactobacillus* decreased over time (AG group: 2.1, 0.7, and 0.2% at 7, 14, and 30 days, respectively; AC group: 5.5, 2.0, and 1.3% at 7, 14, and 30 days, respectively); whereas that of *Streptococcus* increased (AG group: 0.7, 10.8, and 13.4% at 7, 14, and 30 days, respectively; AC group: 2.1, 10.5, and 10.9% at 7, 14, and 30 days, respectively). In comparison, *Klebsiella* remained around 10% in both groups at any time point.

### Diversity Analysis of Gut Microbiota

**Figure 2:**
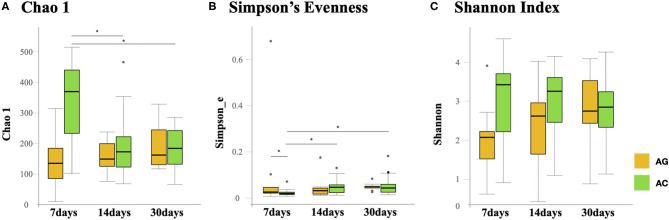
Comparison of gut microbial alpha-diversity in the AG and AC groups on days 7, 14, and 30. **(A)** Richness was assessed using the Chao1 estimator. **(B)** Evenness was assessed using Simpson's Evenness index. **(C)** Diversity was assessed using the Shannon index. AG, ampicillin-gentamicin group; AC, ampicillin-cefotaxime group. Boxes represent the interquartile range, lines indicate medians, and whiskers indicate the range. *Significant differences (*P* < 0.05).

**Figure 3:**
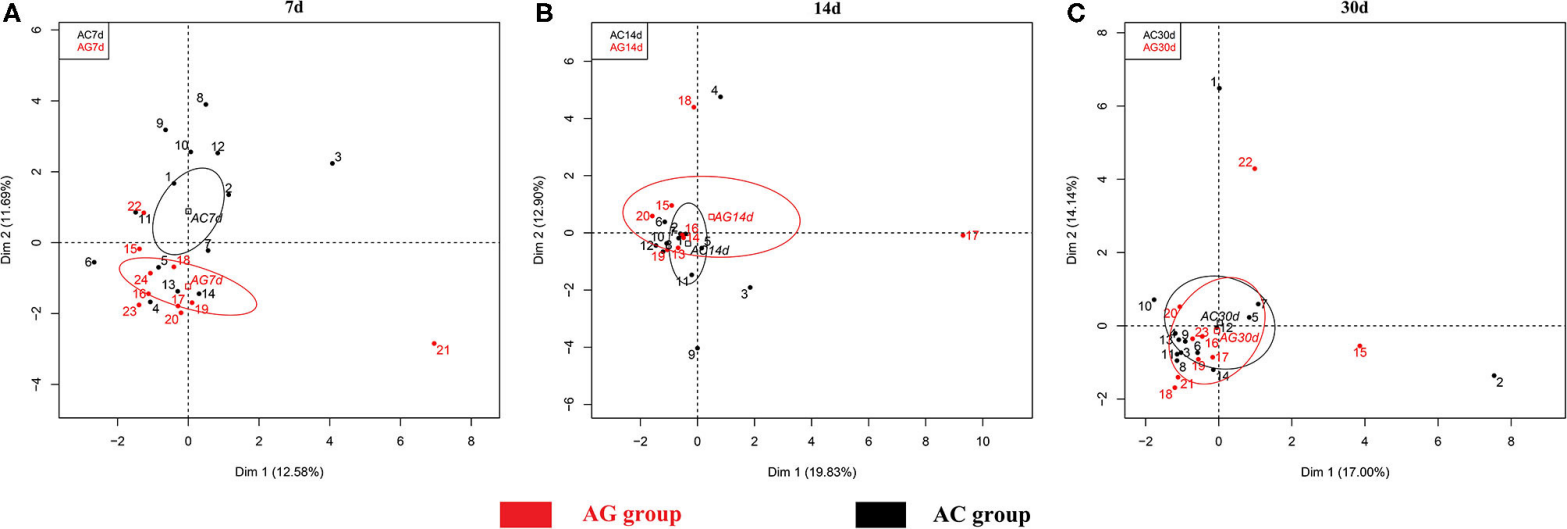
Principal component analysis (PCA) of microbial communities in the AG and AC groups. **(A)** Day 7, **(B)** day 14, and **(C)** day 30. AG, ampicillin-gentamicin group; AC, ampicillin-cefotaxime group.

Species richness (Chao1 index) in the AC group was found to be significantly lower at 14 days (175 vs. 375, *P* = 0.038) and 30 days (185 vs. 375, *P* = 0.03) compared to 7 days (Figure 2A). Microbial evenness (Simpson's E index) was significantly decreased in the AC group compared to that in the AG group (0.02 vs. 0.027, *P* = 0.018) on day 7 (Figure 2B). Moreover, in the AC group, it was lower in the 7-day sample than in the 14-day (0.02 vs. 0.057, *P* = 0.042) and 30-day samples (0.02 vs. 0.053, *P* = 0.042). Differences in richness and evenness were unchanged even after controlling for relevant confounding factors. No significant difference in alpha-diversity (Shannon index) between the two groups was observed over time (Figure 2C). Regarding beta-diversity, although most 7-day AG group samples were well-separated and positioned far from their corresponding AC group samples, microbial composition did not differ significantly between the two groups (*P* = 0.131) (Figure 3A). Samples corresponding to days 14 and 30 in these two groups clustered close to each other and were similar in their microbial composition (*P* > 0.05) (Figures 3B,C).

## Discussion

In the present study, we explored the changes in gut microbiota communities in VLBW preterm neonates, who were administered empirical antibiotic therapy. Among breastfed infants, we found a statistically significant decrease in microbial richness and evenness in those receiving ampicillin and cefotaxime over a period of 7 days compared to those receiving ampicillin and gentamicin over 3 days. Prolonged or intensive use of antibiotics increased the abundance of bacteria from the genus *Enterococcus*. This finding correlates with previous observations on differential gut microbiota colonization in VLBW preterm infants administered distinct antimicrobial agents (13, 14, 22, 23).

Antibiotics can alter the taxonomic, genomic, and functional features of gut microbiota. These dysbioses include loss of keystone taxa, reduced taxonomic diversity, metabolic changes, and growth of pathogenic organisms (24). Several factors must be considered when evaluating the effect of antibiotics on gut microbiota, including the spectrum of antibiotics, dose and duration of treatment, and the level of active antibiotics in the intestinal tract. The duration of antibiotic treatment is an important variable with a significant effect on the composition of gut microbiota and metabolites in preterm infants. Previous studies associated the prolonged use of antibiotics with reduced gut microbial diversity (13, 22, 25, 26). Others reported that exposure of preterm infants to different types of antibiotics might alter microbial composition (14, 23). The spectrum of cephalosporins is broader than that of penicillin, potentially leading to greater interference against the normal microflora (27). Gibson et al. reported that cefotaxime was associated with significantly reduced species richness, whereas gentamicin has no known effects in this respect (12). Previous findings also suggested that antibiotic regimens containing third-generation cephalosporins were more frequently associated with development of antibiotic resistance than regimens with aminoglycosides (11). In the present study, it remains to be determined if the observed gut microbial composition should be attributed to the different duration of antibiotic exposure or the administration of different combinations of antibiotics. Therefore, future studies on microbial composition should compare the outcomes of different antibiotic combinations with the same treatment duration.

Bacterial colonization in the intestine proceeds rapidly after birth, particularly with aerotolerant microbes (phase I bloom) (28, 29). Our study confirms that preterm birth and antibiotic exposure result in inadequate phase I colonization. *Firmicutes* were found to be dominant in the intestinal tract, corroborating previous results on preterm infants (30). In line with previous findings, our study also revealed that neonates exposed to ampicillin and cefotaxime harbored more *Proteobacteria* but fewer *Actinobacteria*, thus causing a shift in the composition of gut microbiota (31, 32). At the genus level, antibiotic treatment in the perinatal period negatively affected the abundance of protective commensal anaerobic bacteria such as *Bifidobacteria* (25, 31, 33, 34). As *Bifidobacteria* have been associated with expression of inflammatory response genes and stimulation of genes that promote integrity of the mucosal barrier, the risk of NEC in preterm infants increases with a decline in *Bifidobacteria*, resulting in an exaggerated inflammatory response (11, 17). Although the abundance of *Bifidobacteria* in this study did not change significantly, their percentage increased over time in the ampicillin-gentamicin group, while decreasing in the ampicillin-cefotaxime group. As shown in this study, prolonged use of antibiotics or third-generation cephalosporin treatment might result in excessive growth of *Enterococcus*. This observation is in line with the results of previous studies (14, 31, 35, 36). An increased abundance of not only *Enterococcus*, but also pathogenic bacteria, such as *Enterobacteriaceae, Streptococcus, Pseudomonas*, and *Clostridium difficile*, has been observed with perinatal antibiotic therapy (13, 14, 30, 31). These findings support the hypothesis whereby antimicrobial agents selectively kill sensitive bacteria and allow rapid growth of antibiotic-resistant strains. According to a recent meta-analysis, neonates exposed to antibiotics carried a higher risk of developing antibiotic-resistant bacteria (11).

Our study did not address the association between differences in gut microbiota and clinical outcomes. It has been shown that short-term use of antibiotics might have a beneficial effect on reducing adverse outcomes in VLBW preterm infants (18, 37, 38). However, a study also highlighted the association between cefotaxime and adverse clinical outcomes (39). Here, such outcomes, including NEC and sepsis, were too few to determine associations with antibiotic exposure. Alterations in the composition of gut microbiota, fewer *Bifidobacteria*, decreased microbiota diversity, and overgrowth of pathogenic bacteria have been linked to NEC and LOS (40–44). Thus, altering microbiome composition through nutrient intervention including probiotics offers a promising strategy for the prevention of NEC and LOS (45, 46). Another unknown in our study is the recovery time for gut microbiota after antibiotic therapy is discontinued. The recovery rate depends on the duration of antibiotic treatment and the regimen applied. Short-term use of antibiotics may have only mild and temporary effects on gut microbiota; whereas infants exposed to antibiotics for a prolonged time harbor a persistently altered microbiota, which may continue up to the age of 3 months (25, 30). In our study, bacterial diversity at 1 month did not differ significantly between the two groups, which correlates with a previous study (22). This finding confirmed the notion that, although bacterial acquisition was perturbed following the use of antibiotics, microbiota continued to populate the gastrointestinal tract of neonates and eventually restored it to its pre-intervention equilibrium.

Among the strengths of our study is the use of serial fecal samples of preterm neonates to compare gut microbiota profiles. The study takes into account also the most significant factor associated with development of the microbiome community structure in early life, breastfeeding (2, 47–49). However, our study has also some limitations. We did not have a control group with no antibiotic exposure because all VLBW preterm infants in our NICU received empiric antibiotics at birth for presumed sepsis. Another limitation was the lack of stool samples collected immediately after birth. Exposure time to the two antibiotic combinations was different, which further limited the scope for comparison between the two groups. As a result, it is unclear, whether the significant findings of our study resulted from the different combination antibiotic therapy or the different duration exposure to the treatment. Moreover, the pre-existence of a higher risk for infection and more premature births in the AC group created a selection bias. We could not control all confounding factors that might have affected gut microbiota composition, such as maternal conditions and diet, gestational age, delivery mode, feeding types, other medications, and nutritional supplements. Further studies controlling these important confounding factors are required. Another important limitation of our study was a relatively small sample size, which might have led to study bias and insufficient power to detect any differences. Furthermore, we only included infants exposed to antibiotics in the first week of life and excluded those who received antibiotics afterward. The continuous use of antibiotics early in life may have a more severe and long-term impact on gut microbiota.

In summary, we were able to demonstrate that early administration of intravenous antibiotics to premature infants during the first postnatal week dramatically affected the development of neonatal intestinal microbiota. Different antibiotic regimens exert different effects on the evolution of gut microbiota in terms of composition, richness, and evenness. Prolonged use of third-generation cephalosporins strengthened the characteristic shift toward pathogenic bacteria, as indicated by excessive growth of *Enterococcus* in neonates treated with cefotaxime. However, these changes were no longer seen at 30 days of age, suggesting that new bacterial genera continued to populate the neonatal gastrointestinal tract despite initial antibiotic exposure. This also implies that rapid cessation of antibiotic treatment may allow for a faster recovery, whereas prolonged use of antibiotics further impairs recovery of the gut microbiota. Although the clinical and long-term health impact of these changes in microbiota remains to be determined, present findings strongly suggest the need to reduce unnecessary use of broad-spectrum antibiotics in preterm neonates. In the future, well-designed large-scale studies should focus on neonates subjected to the same antibiotic regimen, and compare the changes in their gut microbiome before and after antimicrobial therapy. This will help evaluate the effect of antibiotic exposure on gut microbiome metabolites, clinical outcomes, and long-term consequences.

## Data Availability Statement

The datasets presented in this study can be found in online repositories. The names of the repository/repositories and accession number(s) can be found at: NCBI BioProject, accession no: PRJNA659014.

## Ethics Statement

The studies involving human participants were reviewed and approved by Institutional Review Board of MacKay Memorial Hospital according to the 1964 Declaration of Helsinki and its later amendments (IRB number: 17MMHIS026e). Written informed consent was obtained from the relevant individual(s), and/or minor(s)' legal guardian/next of kin to participate in this study.

## Disclosure

The Delta Electronics Incorporation was not involved in the study design, collection, analysis, interpretation of data, the writing of this article or the decision to submit it for publication.

## Author Contributions

H-YC, J-SCC, and H-CL have prepared the project of this study. H-YC, J-HC, C-HH, and C-YLin performed participant recruitment. H-YC, J-SCC, Y-HH, K-NT, C-YLiu, and MK performed data collection and statistics. H-YC, J-SCC, Y-HH, MK, and H-CL have prepared the draft of manuscript. All authors revised and approved the final manuscript.

## Conflict of Interest

Y-HH, K-NT, and C-YLiu were employed by the company Delta Electronics Incorporation. The remaining authors declare that the research was conducted in the absence of any commercial or financial relationships that could be construed as a potential conflict of interest.
